# Supplementary material for: Reconciling Mining with the Conservation of Cave Biodiversity: A Quantitative Baseline to Help Establish Conservation Priorities
Source: PLoS One. 2016 Dec 20;11(12):e0168348. doi: 10.1371/journal.pone.0168348 (PMC5173368; doi:10.1371/journal.pone.0168348)
Supplement: S1 Dataset — (ZIP) [file pone.0168348.s002.zip › Taxa/Serra Norte/SN_2013/N4E/Lista N4E-43.pdf]

CAVIDADE N4E-0043

Classe	Ordem	Fam/Outros	Gên/Outros	Espécie	S-U	U-S	Zon.
Arachnida	Acari	Ixodidae	*Amblyomma*	sp.1		X	E
Arachnida	Amblypygi	Phrynidae	*Heterophrynus*	*longicornis*	1		P
Arachnida	Araneae	Araneidae	*Eustala*	sp.1		X	E
Arachnida	Araneae	Caponiidae	*Nops*	sp.1	X		E
Arachnida	Araneae	Drymusidae	*Drymusa*	*spelunca*		X	E
Arachnida	Araneae	Mimetidae	*Ero*	sp.1	X		
Arachnida	Araneae	Mysmenidae		jovem		X	E
Arachnida	Araneae	Ochyroceratidae	*Ochyrocera*	sp.1	X		E
Arachnida	Araneae	Ochyroceratidae	*Ochyrocera*	sp.3		X	E
Arachnida	Araneae	Pholcidae	*Mesabolivar*	sp.1	X	X	E
Arachnida	Araneae	Segestriidae		jovem		X	E
Arachnida	Araneae	Theridiidae	*Theridion*	sp.1	X	X	E
Arachnida	Araneae	Theridiosomatidae	*Plato*	sp.1		X	P
Arachnida	Opiliones	Stygnidae	*Protimesius*	*gracilis*		1	E
Arachnida	Schizomida	Hubbardiidae	aff. *Rowlandius*	sp.1		X	E
Entognatha	Collembola	Cyphoderidae		sp.1	X		E
Entognatha	Collembola	Paronellidae		sp.1	X	X	E/P
Entognatha	Collembola	Paronellidae		sp.4		X	P
Entognatha	Diplura	Campodeidae		sp.1		X	E/P
Entognatha	Diplura	Projapygidae		sp.1	X	X	E/P
Insecta	Coleoptera	Carabidae	*Gen.n.*	sp.17		X	E
Insecta	Diptera	Chironomidae		sp.	X		
Insecta	Diptera	Dolichopodidae		sp.	X		E
Insecta	Diptera	Limoniidae		sp.		X	P
Insecta	Diptera	Phoridae	Metopininae	sp.		X	P
Insecta	Diptera	Psychodidae	Phlebotominae	sp.		X	E
Insecta	Diptera	Tipulidae	Tipulinae	sp.	X		
Insecta	Heteroptera	Reduviidae	Emesinae	sp.6	1	1	E
Insecta	Heteroptera	Reduviidae	Reduviinae	sp.		2	E
Insecta	Homoptera	Cixiidae		sp.3	X		E
Insecta	Homoptera	Cixiidae		sp.5		X	E
Insecta	Hymenoptera	Formicidae	*Pachycondyla*	sp.2		X	P
Insecta	Hymenoptera	Formicidae	*Camponotus*	sp.1	X		E
Insecta	Hymenoptera	Formicidae	*Eurhopalothrix*	sp.1		X	E
Insecta	Hymenoptera	Formicidae	*Gnamptogenys*	sp.1	X		E
Insecta	Hymenoptera	Formicidae	*Hypoponera*	sp.2	X		E
Insecta	Hymenoptera	Formicidae	*Pachycondyla*	sp.2	X	X	E/P
Insecta	Hymenoptera	Formicidae	*Solenopsis*	sp.1		X	P
Insecta	Isoptera	Termitidae	*Nasutitermes*	sp.	5	X	E
Insecta	Lepidoptera	Noctuoidea		sp.5		1	P
Insecta	Lepidoptera	Tineoidea		sp.3	X		E
Insecta	Orthoptera	Phalangopsidae	*Paraclodes*	jovem	2	5	E/P
Insecta	Orthoptera	Phalangopsidae	*Phalangopsis*	sp.	4	19	E/P
Insecta	Psocoptera	Epipsocidae	*Epipsocus*	sp.4	X	X	E
Amphibia	Anura	Strabomantidae	*Pristimantis*	cf. *fenestratus*	5		E/P
Mammalia	Chiroptera	Emballonuridae	*Peropteryx*	*kappleri*	3	1	P
Mammalia	Chiroptera	Furipteridae	*Furipterus*	*horrens*	1		P
